# Converging lens radiotherapy (CLRT) employing kilovoltage x‐ray source: Treatment planning study

**DOI:** 10.1002/mp.18007

**Published:** 2025-08-08

**Authors:** Marvin Kinz, Adi Alfassi, Ella Gebert, Dror Alezra, Jürgen Hesser, Piotr Zygmanski

**Affiliations:** ^1^ Department of Radiation Oncology, Brigham and Women's Hospital, Dana‐Farber Cancer Institute Harvard Medical School Boston Massachusetts USA; ^2^ Mannheim Institute for Intelligent Systems in Medicine (MIISM), Medical Faculty Mannheim Heidelberg University Mannheim Germany; ^3^ Convergent Radiotherapy and Radiosurgery Ltd. Haifa Israel; ^4^ Interdisciplinary Center for Scientific Computing (IWR) Heidelberg University Heidelberg Germany; ^5^ Central Institute for Computer Engineering (ZITI) Heidelberg University Heidelberg Germany; ^6^ CZS Heidelberg Center for Model‐Based AI Heidelberg University Mannheim Germany

**Keywords:** converging lens radiotherapy (CLRT), non‐coplanar kV x‐ray lens treatment planning, organ at risk (OAR) sparing

## Abstract

**Background:**

Converging lens radiotherapy (CLRT) is a novel radiotherapy modality employing a lens which produces a nearly monoenergetic x‐ray beam at 60 keV to focus radiation on deep‐seated tumors, enabling highly precise dose delivery.

**Purpose:**

This study represents the first comparison of CLRT treatment plan quality to conventional intensity‐modulated radiation therapy (IMRT) and volumetric modulated arc therapy (VMAT). The goal is to provide a proof‐of‐concept of CLRT treatment planning and to evaluate whether CLRT could potentially offer improved organ‐at‐risk (OAR) sparing and target coverage.

**Methods:**

The physical CLRT prototype system was developed by Convergent Radiotherapy and Radiosurgery Ltd. (CRnR) in Haifa, Israel, under the product name *AngelCure*. We initially characterized the CLRT beam model using Monte Carlo simulations, validating it with film dosimetry of the physical beam. Subsequently, we developed a 3D dose computation algorithm and novel inverse, non‐coplanar treatment planning for CLRT inside the open‐source radiation treatment planning platform *matRad*. Two prototype lenses with different focal spot sizes (A ∼11×1 mm and B ∼23×3 mm) were employed. Using these tools, we performed CLRT treatment plan optimization for selected treatment sites and compared our results retrospectively to conventional IMRT/VMAT patient plans from the Dana‐Farber Brigham Cancer Center clinical database, which were generated in *Eclipse* (Varian Medical Systems, Palo Alto, CA, USA). Here, we present three cases from the central nervous system (CNS), thoracic, and genitourinary (GU) sites. We utilized conformity index (CI), target coverage index (TCI), conformal number (CN), and gradient index (GI) as plan quality metrics, together with dose‐volume histogram points to compare OAR sparing.

**Results:**

CLRT demonstrated variable OAR dose sparing. In the CNS case, both lenses showed better hypothalamus sparing, with 0% of the volume receiving 400 cGy or more compared to 91% for Linac. For the Thoracic case, Lens A achieved 0% of esophagus volume receiving more than 1000 cGy, versus 11% for Linac. In the GU case, Lens A delivered at least 400 cGy to 0% of rectum volume, compared to 17% with Linac. CLRT demonstrated potentially better or comparable plan quality metrics, with Lens A showing perfect conformity (CI = 1.00) in the CNS case and Lens B demonstrating the steepest dose gradient (GI = 240%/cm).

**Conclusions:**

This study serves as a first proof‐of‐concept of the treatment capabilities of a novel CLRT x‐ray system. The system demonstrated, in this limited set of plans, better OAR dose sparing compared to conventional Linac‐based plans across 3 different anatomical sites, accompanied by similar, and possibly better, plan quality metrics (CI, TCI, CN, GI), while maintaining comparable target coverage.

## 1 INTRODUCTION

Linear accelerator (Linac)‐based methods, including intensity‐modulated radiation therapy (IMRT) and volumetric modulated arc therapy (VMAT), have advanced the field of cancer treatment. Nonetheless, sparing of critical organs‐at‐risk remains a persistent challenge.^1^ In response to this challenge, converging lens radiotherapy (CLRT) has been developed by Convergent Radiotherapy and Radiosurgery Ltd., Haifa, Israel (CRnR).^2^, ^3^

The CLRT system, under development as AngelCure at CRnR, is a frameless robotic radiotherapy platform that delivers highly focused kilovoltage (kV) radiation. The system integrates a conventional kV‐range x‐ray tube with a proprietary focusing lens, generating a convergent, hollow, and nearly monoenergetic beam at approximately 60 keV. A fixed internal collimator, positioned between the x‐ray tube and the lens, restricts the beam to the area of the focusing lens, which then directs the photons to the focal volume. As a result, the diverging beam from the x‐ray source converges to a predefined volume. Mounted on a 6‐degree‐of‐freedom robotic arm, the *AngelCure* system combines radiation delivery with imaging and patient support, enabling precise external beam targeting. The system will be designed to treat target volumes up to 24 cc with a maximum penetration depth of 8 cm to the target center in normal tissue, and deeper in lung anatomy. CLRT thus has the potential to enable low‐cost design of treatment rooms with minimal shielding, low‐footage, and low‐power requirements.

The potential for lower total cost of ownership was recognized by other groups as well, and thus the development of keV and convergent x‐ray sources for radiotherapy of deep‐seated tumors has been an area of active research in recent years.^4^ For instance, O'Connell et al.^5^ developed a non‐coplanar treatment approach for stereotactic ablative radiotherapy using keV treatment arcs, aimed at low‐ and middle‐income countries. Another arc‐based system has been simulated, which utilizes a collimator to create an array of converging keV beamlets.^6^ Additionally, research into polycapillary x‐ray optics have demonstrated the feasibility of creating focused keV beams for localized dose delivery.^7^ Another benefit of keV is the higher dose deposition in contrast‐enhanced radiotherapy, which led Loughery et al.^8^ to present proof‐of‐concept of a keV IMRT system.

To evaluate the normal organ sparing capabilities of the novel CLRT technology, we first modeled the CRnR source and developed dose computation and treatment plan optimization algorithms. Subsequently, we performed CLRT treatment planning optimization for selected treatment sites and compared the results to Linac‐based IMRT/VMAT plans, which have been delivered at the Dana‐Farber Brigham Cancer Center.

## 2 METHODS

For treatment planning purposes, a fully functional radiation source and two prototype versions of the CRnR lens were employed. They are both characterized by two key features: a quasi‐monoenergetic photon energy of 60 keV and a hollow conical convergent beam geometry focusing on small focal volumes of various sizes:

- **Lens A**: Features an elongated beam focal volume with dimensions of approximately 22.5 mm FWHM (full width at half maximum) in the longitudinal direction and 3.4 mm FWHM in the lateral direction in water.
- **Lens B**: Exhibits a more compact beam focal volume, measuring about 10.8 mm FWHM longitudinally and 1.3 mm FWHM laterally in water.

This converging beam geometry allows for highly localized dose delivery, with a pronounced peak dose at the focal spot and lower doses in the surrounding converging radiation cone. In contrast to the converging x‐ray beam of CRnR, a conventional Linac produces a divergent beam. For highly focused treatments such as stereotactic radiosurgery (SRS), stereotactic radiotherapy (SRT), and stereotactic body radiotherapy (SBRT), the lateral FWHM of the beam can be as small as approximately 3 mm, achieved through high‐definition multi‐leaf collimation. However, to achieve a 3D focusing effect, the Linac beam must be rotated around the tumor center using gantry and couch rotations. While couch rotations are feasible for brain tumors, they are typically not used for other treatment sites due to clearance issues. Consequently, the focusing effect for small tumors in Linac‐based treatments may not be as sharp as that of the CRnR beamline, which benefits from double focusing via an x‐ray lens and unrestricted robotic rotations.

To utilize CLRT, custom in‐house dose computation and inverse treatment planning algorithms were developed. Doses in water and heterogeneous media were computed using Monte Carlo simulations and validated by using film dosimetry of the physical beam.^3^ For treatment planning, a fast dose computation algorithm was developed to compute doses in heterogeneous patient CTs based on the Monte Carlo data. The supplemental material covers the validation process and methodology.

**FIGURE 1:**
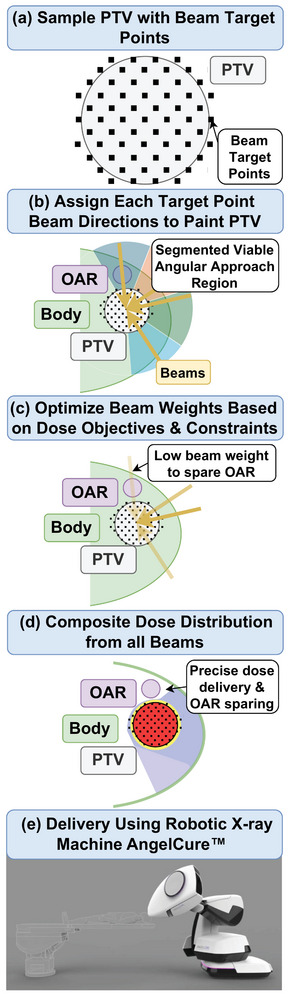
Treatment planning process.

The inverse treatment plan optimization utilized a modified version of *matRad*,^9^ an open‐source software for radiation treatment planning based on *MATLAB R2023b* (MathWorks, Natick, MA, USA), to generate comparison plans for the novel convergent beamline. Given the beamline's unique features, we developed a custom non‐coplanar treatment approach to target dose painting with a robotic arm. Figure 1 gives an overview of the planning process. It consists of several key steps:

- **Tumor volume sampling**: The target volume is sampled with beam target points, which represent the location of focal volumes within the tumor. They are spaced slightly wider than the beam's lateral FWHM, ensuring complete dose coverage. Each of these points is potentially irradiated from different directions. However, beam directions are restricted in the subsequent beam direction selection step.
- **Beam direction selection**: The viable angular approach region for the target points is determined, considering the tumor's depth and the fixed converging beam geometry. This describes the 3D angular space of possible beam directions for each target point. The maximum viable depth is limited by the distance between the treatment head exit and the beam focal volume. Within this region, concrete beam directions are assigned to each target point in the next step.
- **Target point angle assignment**: The viable approach region is segmented into parts of equal size, and each target point is assigned one angle from each segment. This approach aims to diversify potential directions for each target point. In the context of this study, the number of segments was limited by computing power. After setting up all beam targets and directions, the optimal beam weights are calculated.
- **Beam weight optimization**: Inverse optimization of the beam weights w follows the existing framework supplied by *matRad*.^9^ Dose objectives are assigned to the PTV and OARs in the form of squared under‐ and over‐dosing, which were found to lead to the best results.
(1) $$\begin{array}{cc} f_{\text{sq ud}} & =\frac{1}{N_{S}}\Sigma_{i\varepsilon S}\Theta \left(\hat{d}-d_{i}\right){\left(d_{i}-\hat{d}\right)}^{2}\\ f_{\text{sq od}} & =\frac{1}{N_{S}}\Sigma_{i\varepsilon S}\Theta \left(d_{i}-\hat{d}\right){\left(d_{i}-\hat{d}\right)}^{2} \end{array}$$
  Θ(x) represents the Heaviside function, $d_{i}$ the dose in voxel i, $\hat{d}$ the prescribed dose, S the structure, and $N_{S}$ the number of its voxels. These functions $f_{n}$ are combined with a penalties $p_{n}$ to a weighted sum, which is then minimized.
(2) $$\begin{array}{cc} & \min_{w\in R^{B}}f\left(w\right)=\sum_{n}p_{n}f_{n}|\left(w\right)\\ & \;\text{subject to}\;\;c_{k}^{l}\leq c_{k}\left(w\right)\leq c_{k}^{u},0\leq w, \end{array}$$
  where $c_{k}\left(w\right)$ is the k‐th constraint with upper and lower bounds.
- **Dose distribution**: The final dose distribution is calculated by accumulating the weighted beams post‐optimization
(3) $$d_{i}=\sum_{j}D_{ij}w_{j}\;,$$
  where $D_{ij}$ is the dose matrix for all voxels i and beams j.

This customized approach leverages *matRad*'s modular design and *MATLAB*‐based architecture, allowing for efficient integration of the novel beamline's characteristics into the treatment planning process. The non‐coplanar beam configuration is particularly advantageous for complex target geometries, potentially offering improved dose conformity and OAR sparing compared to traditional coplanar techniques.

The plans chosen for comparison were sourced under retrospective IRB approval from actual cases in the Dana‐Farber Brigham Cancer Center clinical database that have been delivered for treatment using a Linac and were planned using *Eclipse* (Varian Medical Systems, Palo Alto, CA, USA) as IMRT or VMAT. For re‐planning using CLRT, the structures and prescription were kept as they were for the actual plan that had been delivered, and OARs relevant to the case were spared by using fitting constraints.

To compare the plans, we utilized the dose volume histograms (DVHs) of the OARs to estimate the dose sparing and used multiple standard indices to evaluate the dose delivery. The conformity index (CI) describes how well the dose conforms to the target, the target coverage index (TCI) is a measure for target dose coverage, and the conformal number (CN) is the product of the two. For all indices, a value closer to one indicates better conformity or coverage. The gradient index (GI) assesses the steepness of the dose fall‐off outside the target volume in %/cm, thus a higher value is desirable.^10^

## 3 RESULTS

Table 1 gives an overview of the three plans chosen from the Dana‐Farber Brigham Cancer Center clinical database for comparison. They cover diverse sites, including the central nervous system (CNS) in Case 1, thoracic in Case 2 and genitourinary (GU) in Case 3, and have been delivered between 2021 and 2023. Prescribed dose varies between 27 and 40 Gy, planning target volumes (PTVs) vary between 0.36 and 3.64 cc, and the energy was 6X FFF (6 MV Photon Flattening Filter Free) for all cases. The table also contains multiple indices for dose delivery plan comparison between the different modalities, which will be discussed in the following sub‐sections on a per case basis.

**TABLE 1:** Overview of treatment case details and plan comparisons including the conformity index (CI), the target coverage index (TCI), the conformal number (CN), and gradient index (GI).

Case details	Case 1			Case 2		Case 3	
Site	CNS‐Mets			Thoracic‐NSCLC		GU	
Planned dose $D_{plan}$	27 Gy			34 Gy		40 Gy	
Number fractions	5			1		5	
Volume PTV $V^{PTV}$	0.36 cc			3.64 cc		2.98 cc	
Linac energy	6X FFF			6X FFF		6X FFF	
Linac technique	VMAT SRT			VMAT SBRT		IMRT Adapt. SBRT	
**Plan comparison**	Linac	Lens A	Lens B	Linac	Lens A	Linac	Lens A
CI ($V_{100}^{PTV}/V_{100}$)	0.92	**1.00**	0.89	**0.89**	0.74	0.73	**0.92**
TCI ($V_{100}^{PTV}/V^{PTV}$)	**0.99**	0.98	0.97	**0.97**	**0.97**	0.86	**0.92**
CN (CI × TCI)	0.91	**0.99**	0.87	**0.87**	0.72	0.63	**0.85**
GI ($\frac{50\%}{R_{50}^{eff}-R_{100}^{eff}}$) ($\frac{\%}{cm}$)	111	181	**240**	58	**67**	**84**	79

*Note*: $V_{x}$ and $R_{x}^{eff}$ describe the volume and effective radius of x percent of the planned dose $D_{plan}$, respectively. Bold indicates the best value in each comparison.

Bold indicates the best value in each comparison.

### 3.1 Case 1: CNS‐Mets

Case 1 focuses on the treatment of a single metastasis in CNS, utilizing a planned dose of 27 Gy delivered in 5 fractions. This case involves a small PTV volume of 0.36 cc positioned at the cranial part of the brainstem and employed VMAT SRT as a technique.

**FIGURE 2:**
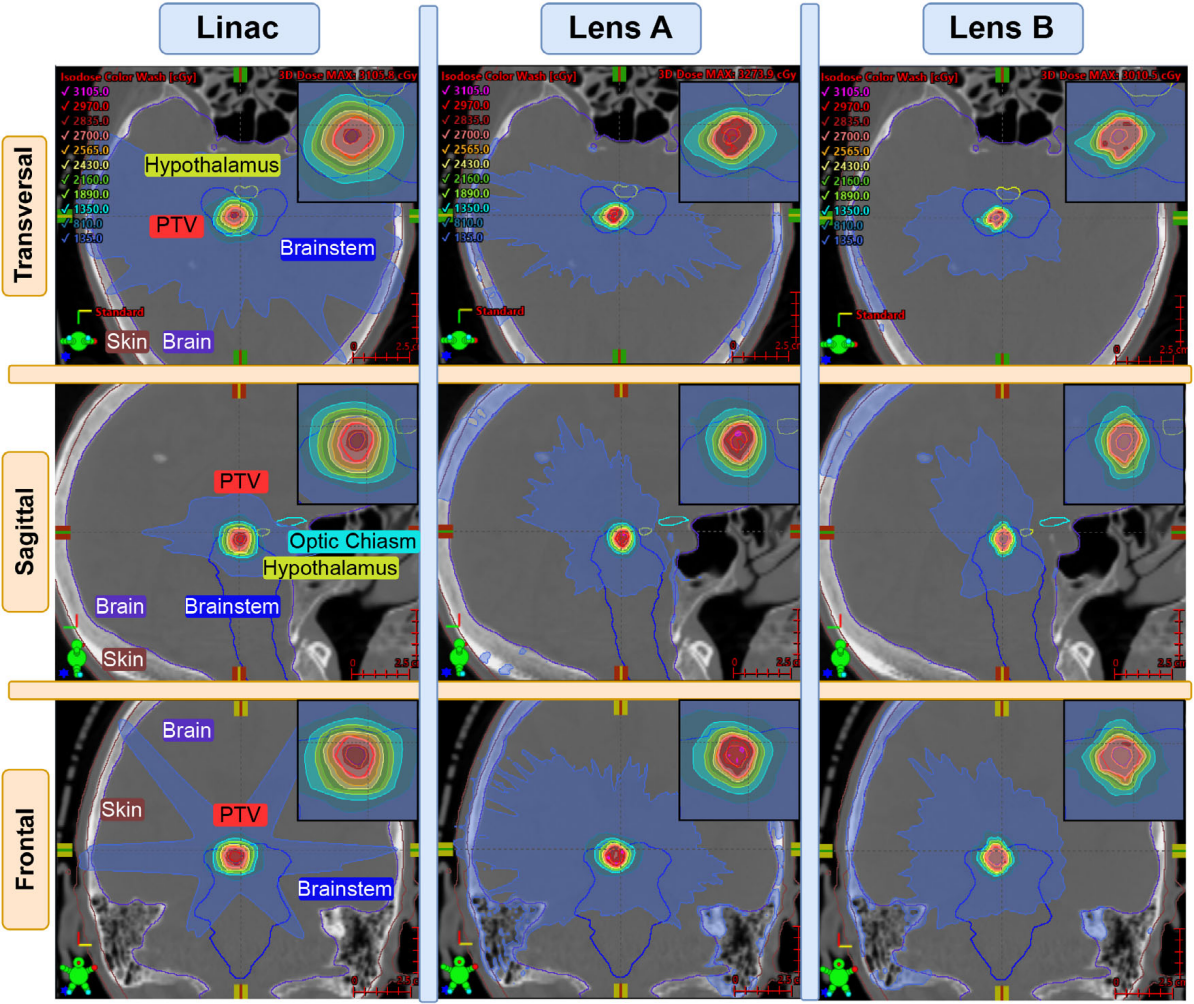
Plan comparison for Case 1. The columns show the different treatment devices, and the rows show different views. The PTV is contoured in red, which is mostly overlapping with the salmon color of the planned dose at 27 Gy. A zoom on the PTV is provided in the upper right corner of every image. PTV, planning target volume.

Figure 2 presents a comparative analysis of dose distributions between the original Linac plan and two versions of the convergent lens. The frontal view of the Linac plan displays a characteristic dose pattern resulting from a single VMAT arc encompassing the head and two short arcs above it. In contrast, both CLRT plans demonstrate a more uniform dose distribution around the target, as they approach the target from additional access angles. They also show a disconnected low‐dose region far from the PTV, in the skull, which is due to the higher photoelectric absorption of the 60 keV beam in bone, compared with the Linacs MV beam. Examining the dose fall‐off around the PTV reveals that Lens A exhibits a steeper gradient than the Linac plan, while Lens B demonstrates an even tighter fall‐off. This translates to reduced dose delivery to healthy tissue surrounding the PTV in the convergent lens plans compared to the Linac‐based plan.

**FIGURE 3:**
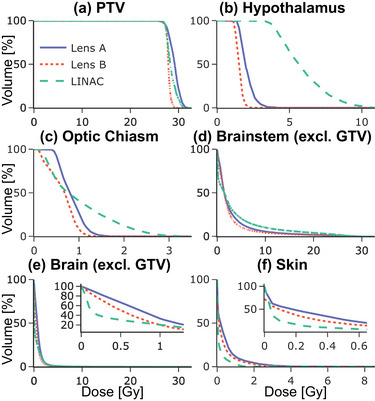
Case 1 DVHs for PTV and OARs. DVHs, dose volume histograms; OAR, organ‐at‐risk; PTV, planning target volume.

Figure 3 includes DVHs describing the dose coverage for the target and dose sparing for each OAR.

### 3.2 Case 2: Thoracic‐NSCLC

Case 2 addresses the treatment of thoracic non‐small cell lung cancer (NSCLC), with a planned dose of 34 Gy delivered in a single fraction using VMAT SBRT. This case involves a larger PTV volume of 3.64 cc situated in the upper left lung.

**FIGURE 4:**
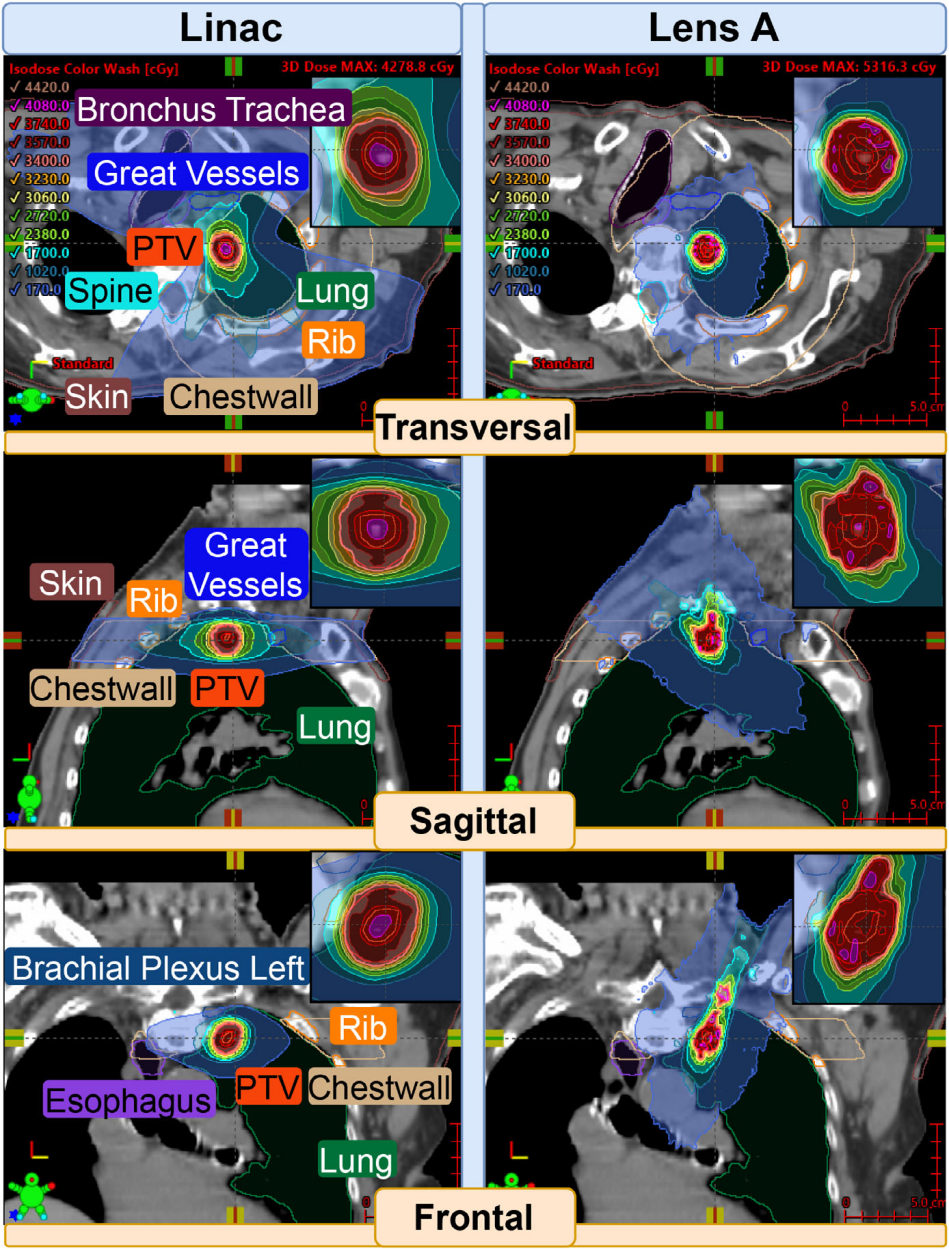
Plan comparison for Case 2.

Figure 4 illustrates the dose distributions generated by the Linac and the novel converging x‐ray lens across multiple views. The Linac plan employs a typical VMAT technique, utilizing two half arcs in a single plane. In contrast, the CLRT based plan delivers most of the dose through the shoulder region, which is particularly evident in the sagittal and frontal views. The dose distribution characteristics reveal that the lens achieves a tighter dose distribution in the transversal plane compared to the Linac, while both methods demonstrate comparable distributions in the sagittal view. However, in the frontal view, the Linac plan exhibits a superior dose distribution relative to the lens.

**FIGURE 5:**
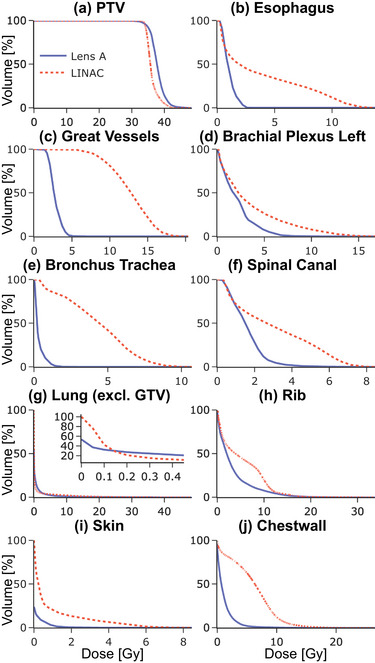
Case 2 DVHs for PTV and OARs. DVHs, dose volume histograms; OAR, organ‐at‐risk; PTV, planning target volume.

Figure 5 presents the DVHs for OARs, highlighting the CLRTs enhanced sparing capabilities across all assessed OARs when compared to the original Linac plan.

### 3.3 Case 3: GU

Case 3 is centered on genitourinary (GU) radiotherapy, with a planned dose of 40 Gy delivered in 5 fractions using IMRT adaptive SBRT. This case involves a PTV volume of 2.98 cc located close to the large bowel in the lower right abdomen.

**FIGURE 6:**
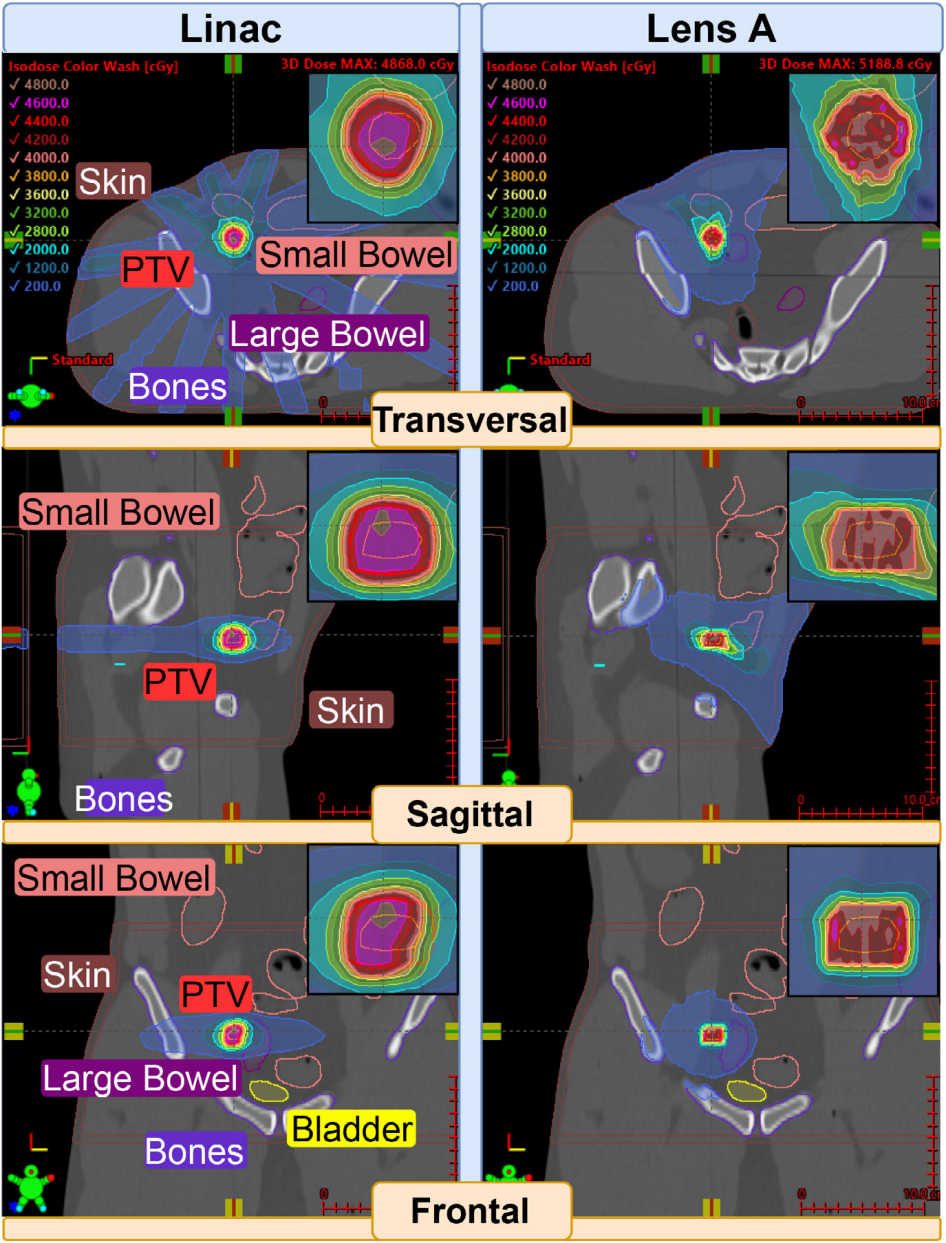
Plan comparison for Case 3.

Figure 6 shows the dose distribution. This is an adaptable MR Linac plan, which is why VMAT could not be used, and instead the typical IMRT streaks are clearly visible in the transversal view of the linac plan. The lens, on the other hand, approaches the tumor frontally from a large region in the right lower abdomen. This leads to a very tight dose distribution in the frontal direction, while being less tight, but still comparable to the IMRT plan, in the other two directions.

**FIGURE 7:**
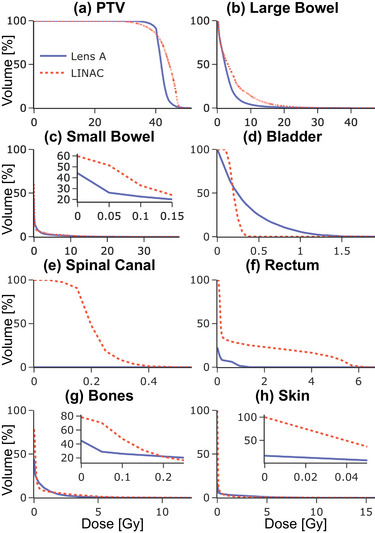
Case 3 DVHs for PTV and OARs. DVHs, dose volume histograms; OAR, organ‐at‐risk; PTV, planning target volume.

Figure 7 gives an overview over the DVHs of the PTV and the OARs. The lens delivers a more homogeneous dose to the target and less dose to most of the OARs.

## 4 DISCUSSION

In the CNS case, the results illustrate large dose reductions to the hypothalamus, optic chiasm, and brainstem for both lenses compared to the VMAT plan. Whole brain and skin receive increased low‐dose coverage from the two lenses. Even so, only 7.77% of the normal brain volume received more than 150 cGy with Lens B, compared to 11.95% for Linac and 14.97% for Lens A. Both CLRT plans outperformed the Linac plan in brainstem protection, with the volume of normal brainstem receiving 1000 cGy or more being lower for Lens A (5.76%) and Lens B (4.32%) compared to Linac (10.3%). Perhaps most striking is the difference in hypothalamus sparing. While 91.35% of the hypothalamus volume received at least 400 cGy with Linac, this drops to 0% for CLRT. All plans keep the D50 well below the 20 Gy threshold associated with increased risk of hormone deficiency, but the CLRT plans deliver lower dose by a factor of up to three (193.7 cGy for Lens A and 148 cGy for Lens B) compared to Linac (565.64 cGy), potentially further reducing the risk of endocrine dysfunction.^11^ That being said, all OARs are well below the dose constraints set in TG‐101,^12^ if any. The plan quality metrics provide additional insights. Lens A achieves perfect conformity with a conformity index (CI) of 1.00, compared to 0.92 for Linac and 0.89 for Lens B. The conformal number (CN) further emphasizes the advantage of Lens A (0.99) over Linac (0.91) and Lens B (0.87).

Lens B exhibits the highest gradient index (GI) of 240%/cm, suggesting a steeper dose fall‐off outside the target, which may contribute to its superior normal tissue sparing.

The thoracic case also showcases good OAR sparing. For the esophagus, Lens A achieves 0% of the volume receiving 1000 cGy or more, compared to 11.45% for Linac, well within the proposed constraint of V10<30% ^13^ or V11.9 < 5 cc of TG‐101. Lens A also outperforms Linac in bronchus and trachea protection, with a D4cc of 67.5 cGy compared to Linac's 694.7 cGy, although that is also still far from the 10.5 Gy set in TG‐101. Spinal canal protection is superior with Lens A (D1cc: 265.4 cGy) compared to Linac (639.2 cGy), both well below the UK consensus guideline of 7 Gy.^14^ In general, all organs are well below the constraints set in TG‐101. The plan quality metrics reveal that the Linac plan achieves better conformity compared to Lens A (CI: 0.89 vs. 0.74). This discrepancy can be attributed to the lens system's smaller clearance to the patient, which limited the CLRT system to approach the deep‐seated tumor from a single entrance region between neck and shoulder. Consequently, this led to dose build‐up through beam overlap, compromising the overall conformity of the treatment plan.

However, Lens A still achieves a slightly steeper dose gradient (GI: 67%/cm vs. 58%/cm), which aligns with its improved OAR sparing capabilities.

The GU case demonstrates better large bowel protection, with only 1.67% of the selected volume receiving more than 1500 cGy, compared to 5.75% for Linac. This value is clinically interesting, as the V15 of the large bowel is associated with grade 3 CTCAE toxicity.^15^ Rectum sparing is also notably better with Lens A, which delivers at least 400 cGy to 0% of the rectum volume, compared to 16.59% with Linac, although both are well within the limit of V25 < 20 cc set in TG‐101. Lens A shows superior performance in most plan quality aspects, including better conformity (CI: 0.92 vs. 0.73), target coverage (TCI: 0.92 vs. 0.86), and overall plan quality (CN: 0.85 vs. 0.63) compared to the Linac plan. The Linac plan has a slightly steeper dose gradient (GI: 84%/cm vs. 79%/cm).

The ability of CLRT to achieve tight dose distributions and precise OAR sparing, particularly for critical structures near the target, demonstrates the potential advantages of this novel approach. However, this research also acknowledges the preliminary nature of these findings. While the study indicates that tight dose distributions and OAR sparing can likely be achieved using CLRT, it cannot demonstrate direct improvements in clinical outcomes. This limitation arises from the fact that all Linac plans presented here were delivered in our clinic, which already adheres to the common dose restrictions like TG‐101.

Given that the CLRT beam is in the shape of a converging, mostly hollow cone, future studies comparing it to the diverging yet collimated Linac should explore whether this novel platform could provide an alternative to plaque brachytherapy, the current leading solution for ocular melanoma, which is an invasive procedure. Additional areas of future research could include functional ablation for trigeminal neuralgia, ablation of arteriovenous malformations, and possibly ablation of refractory epilepsy, which is currently treated with minimally invasive ablative methods.

In this study, we deployed two prototype lenses and preliminary planning software to give a proof‐of‐concept. While the in‐house TPS was validated using dosimetry films and Monte Carlo OpenGATE simulations for simple geometries, as shown in the supplemental material, it can not be as precise as a purely Monte Carlo‐based dose calculation, especially for heterogeneities. Given the non‐coplanar optimization, using Monte Carlo on the patient CT for all individual beams was too computationally expensive at this preliminary stage. Further development of planning tools that incorporate current state‐of‐the‐art optimization and dose calculation engines is expected. Future studies can focus on further optimizing lens geometric configurations, developing more sophisticated dose painting and optimization techniques, expanding validation across more cancer sites, and comparing more cases for each site so that statistically significant statements can be made.

## 5 CONCLUSION

This study gave a proof‐of‐concept for the novel CLRT system, which utilizes a quasi‐mono‐energetic 60 keV x‐ray lens that generates a hollow conical converging beam and is mounted on a robotic arm. Using this unique beam geometry with a small focal volume and lower‐energy photons, the CRnR lens system potentially offers a promising alternative to conventional linear accelerator‐based treatments.

Comparative analysis in three distinct clinical scenarios, CNS, thoracic, and genitourinary, revealed similar, and potentially superior, organ‐at‐risk‐sparing capabilities, considering the uncertainties associated with the prototype lenses and treatment planning system. The novel lens technology demonstrated promising potential for low radiation exposure to critical structures while maintaining effective target coverage.

## CONFLICT OF INTEREST STATEMENT

The authors have no relevant conflicts of interest to disclose.
